# Anorectal Myiasis Caused by Cochliomyia hominivorax in a Septic Tank Worker

**DOI:** 10.7759/cureus.90842

**Published:** 2025-08-23

**Authors:** Luis A Licona, Pedro M Laud, Reyna F Argueta

**Affiliations:** 1 Family and Community Medicine, 0101 Medicina General, La Ceiba, HND; 2 Surgery, General Hospital of Atlantida, La Ceiba, HND

**Keywords:** cochliomyia hominivorax, endemics central america, myiasis, occupational disease, perianal infestation, screwworm, septic tank worker, submucosal hemorrhoidectomy, tropical parasitosis

## Abstract

The term "myiasis" refers to the parasitic infestation by fly maggots in human tissue. Although cutaneous involvement is well documented, anorectal myiasis is exceedingly rare, particularly when caused by *Cochliomyia hominivorax*, The New World screwworm fly.

We present the case of a 55-year-old male septic tank worker from a rural northern community of Honduras. The patient presented with hyperglycemia, perianal pain, discharge, and prolapsed hemorrhoids. Upon admission, an early evaluation led to a new diagnosis of diabetes type II. We arranged a surgical exploration in the operating theater under general anesthesia. During debridement and exploration, we found numerous larvas invading the anal mucosa and second-/third-degree hemorrhoids.

The treatment included submucosal hemorrhoidectomy, larval extraction, systemic antibiotics, outpatient wound care, glycemic control, and a dose of ivermectin, all of which led to a full recovery.

This case highlights the diagnostic and therapeutic challenges of myiasis in atypical anatomical sites, impaired wound healing in diabetic patients, and emphasizes occupational risk in low-sanitation environments.

## Introduction

Myiasis is a parasitic infestation of human tissue by fly larvas. The most common form of myiasis is cutaneous, though oral, urogenital, stoma site, and gastrointestinal cases have been described as in the article by Das et al. [1]. According to Centers for Disease Control and Prevention (CDC), human anorectal myiasis is extremely rare [2]. Scarce reports implicate *Cochliomyia hominivorax*, an obligate parasite of warm blooded species endemic to Central and South America, as reported by Costa-Júnior et al. [3]. Usually, screwworm flies deposit their larvas directly into open wounds. However, larvas can also penetrate directly into mucosas, such as reported in this and other cases [3-6]. Larvas develop by directly burrowing and feeding from the host’s tissues with their sharp mouth hooks. This generates significant tissue inflammation and, in some cases, a bacterial superinfection. This was especially significant in our case due to the involvement of the anorectal mucosa.

As a special mention, larvas or eggs of the fly may be ingested inadvertently by people and survive the unfavorable conditions of the gastrointestinal tract. However, this presentation is most common with other species of flies such as *S. haemorrhoidalis *as described in by Udgaonkar et al. [6]. 

Remarkable risk factors include living or traveling to endemic areas, poor hygiene, close contact with fecal matter or livestock, exposed open wounds, impaired wound healing, regular use of latrine pits, low educational level, and occupational exposure. Recent reports by local and international epidemiology regulation entities have reported a screw worm outbreak in Central America and Mexico [7,8]. The current outbreak saw 1,207 new animal cases reported in Honduras during the first quarter of 2025. Given the rarity of *C. hominivorax *myasis in humans, official reports of current human cases and mortality are uncommon and not reliable.* *Here, we describe an unusual case of anorectal myiasis caused by *C. hominivorax* in a 55-year-old diabetic man working in septic tank maintenance.

## Case presentation

A 55-year-old male septic tank worker from a rural community in northern Honduras presented to our Emergency department with a three-day history of anal insidious pain, fever, defecation urgency, and dyschezia. The patient's pain upon admission was scaled at 9/10 on the Visual Analog Pain score. Examination revealed an ulcerated red anal mass at 12 o’clock of the anal verge of approximately 1 cm, along with Grade II-III prolapsed hemorrhoids. The mass was painful to touch and had a fairly firm consistency. Additionally, we noticed active laminar bleeding with concomitant whitish malodorous discharge.

His past medical history was remarkable for chronic constipation, hemorrhoids with associated bleeding, newly diagnosed type 2 diabetes mellitus, and intermittent asthma. The patient denied receptive anal intercourse and reported a sexual history limited to vaginal intercourse with women. He had no history of sexually transmitted diseases or trauma. Our patient’s occupational risk factors were significant, as he performed regular informal septic tank maintenance without the use of optimal personal protective equipment. 

Vital signs at presentation at our emergency department were stable. Laboratory findings included mildly elevated white blood cell (WBC) count: 9,450/mm³ (neutrophilic), C-reactive protein: 24.4 mg/dl, HIV negative, and fasting blood glucose of 139 mg/dl.

**Figure 1:**
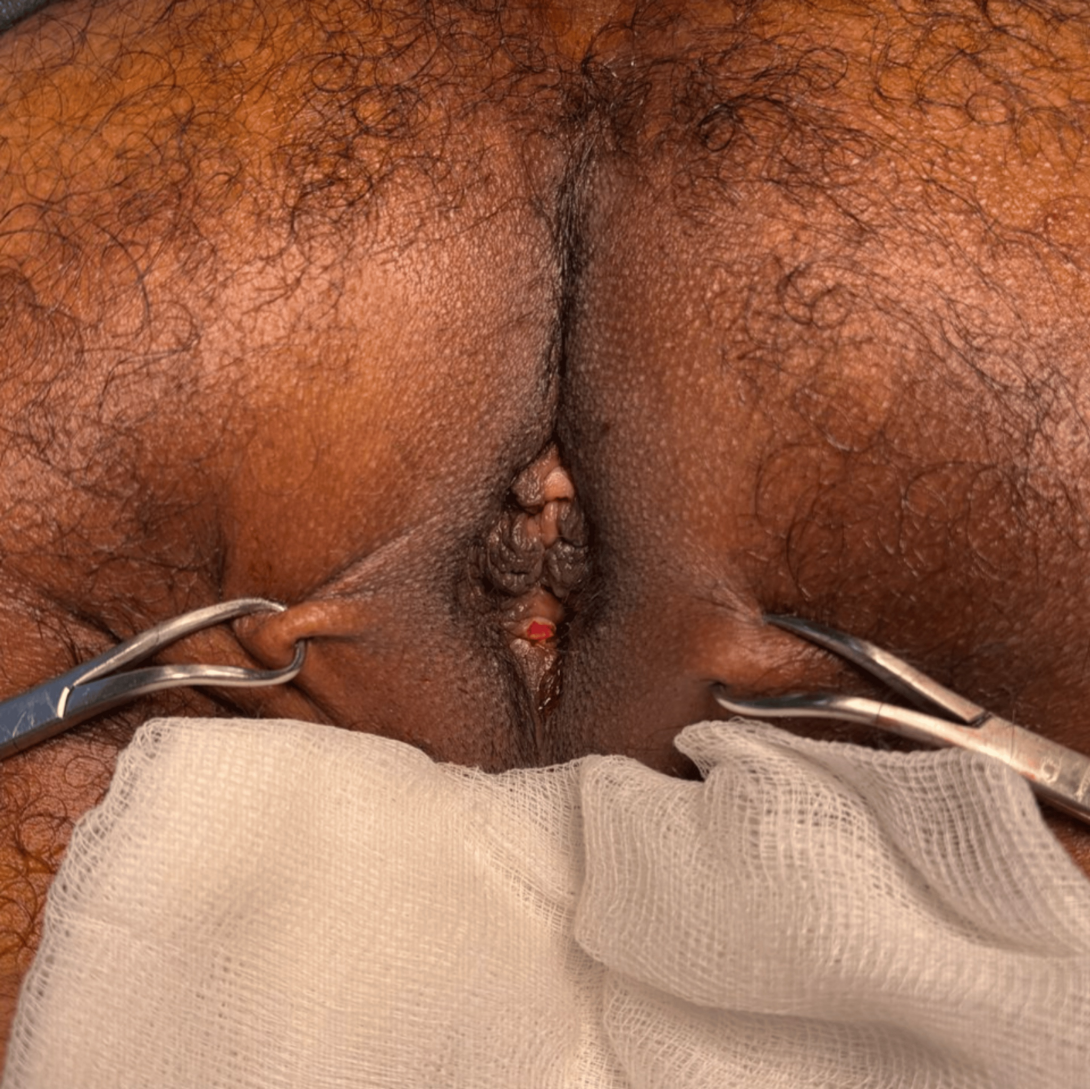
Ulcerated anal lesion at 12 o'clock. The patient is lying in a jackknife position.

**Figure 2:**
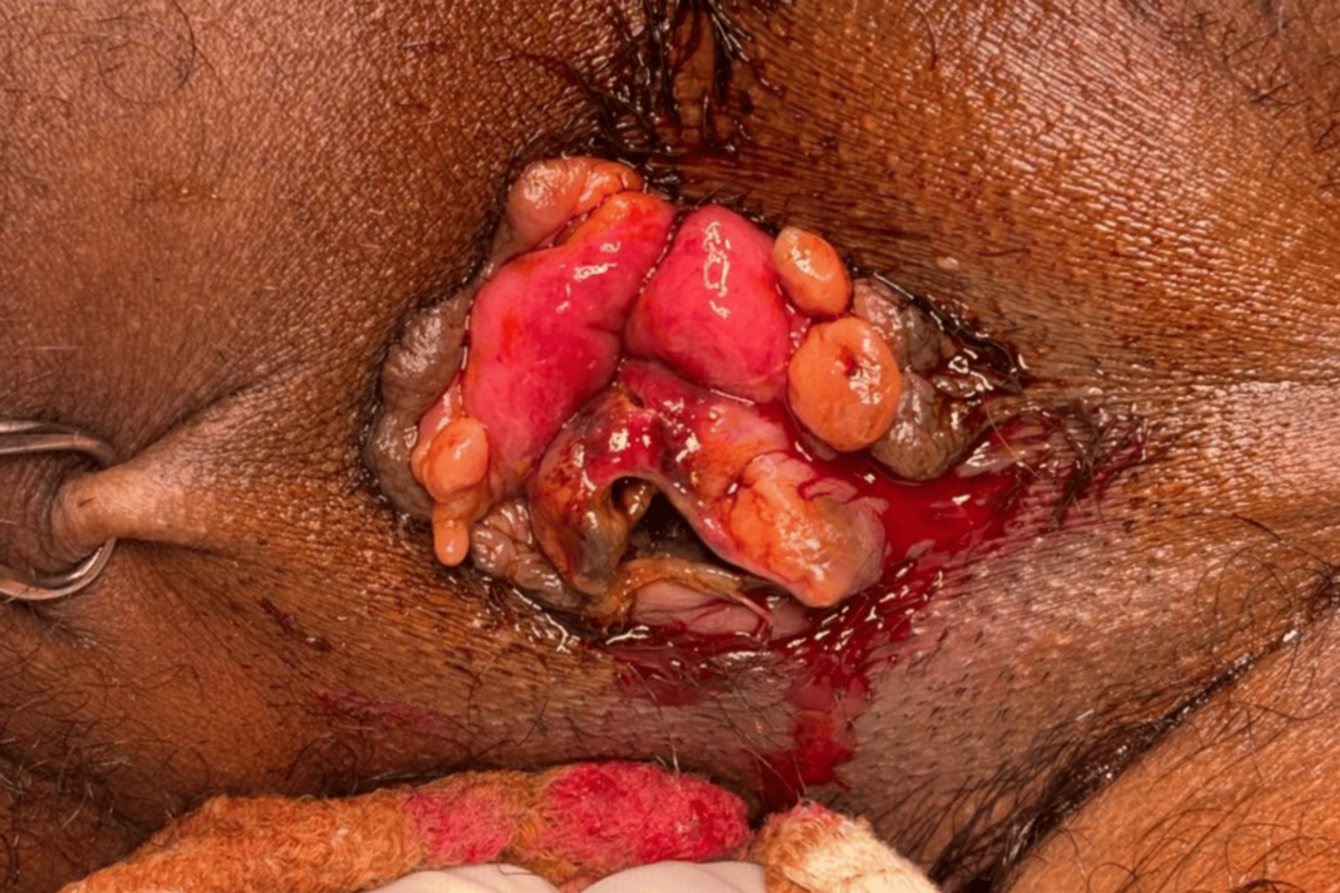
Initial debridement into the anorectal submucosal

**Figure 3:**
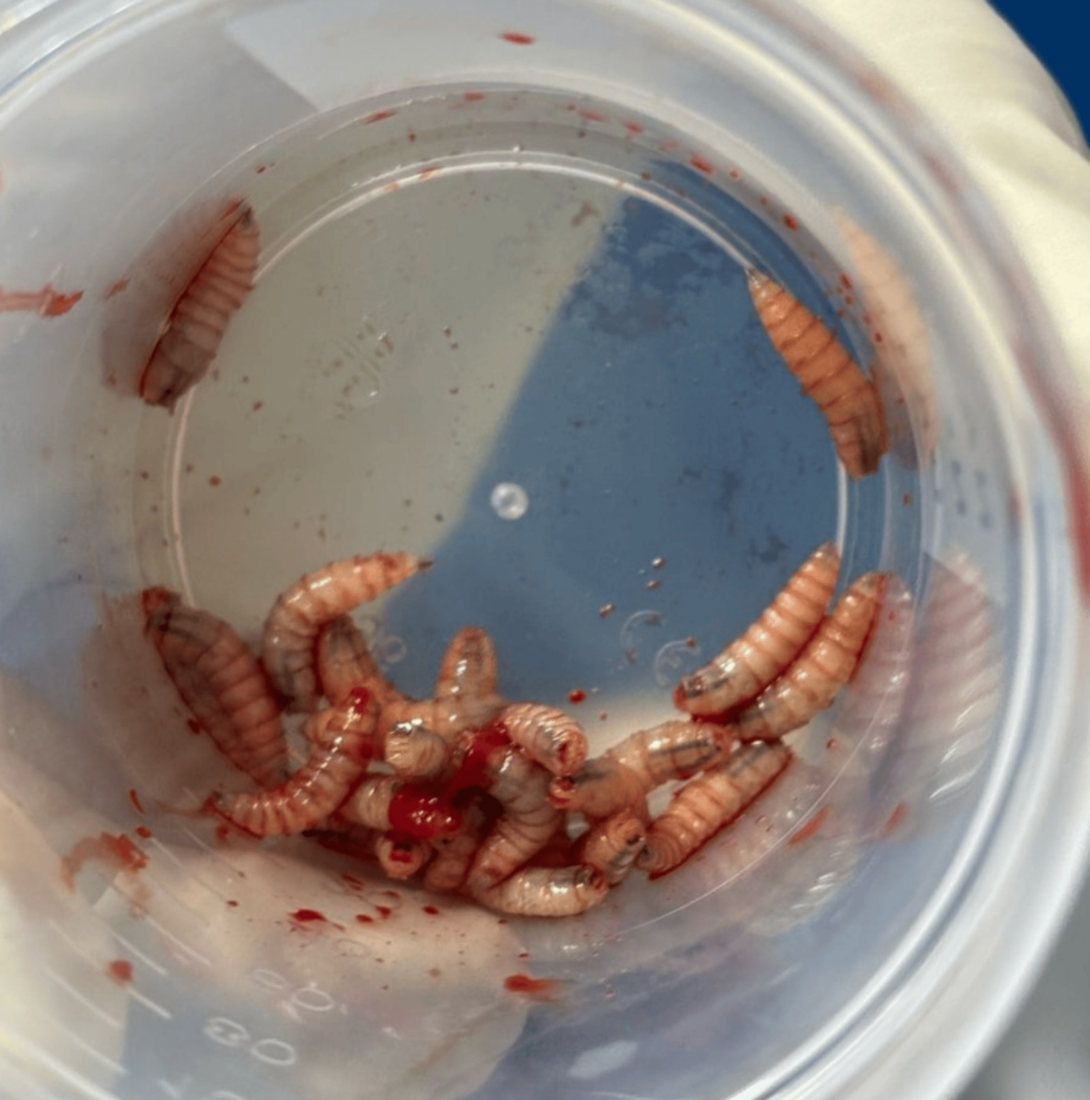
Extracted larvas Larvas identified as *C. hominivorax* by morphology.

Pain limited our initial examination, thus we arranged a surgical exploration under general anesthesia in the operating theater. We administered a single perioperative dose of ceftriaxone 1 g and metronidazol 500 mg IV. The patient was draped and prepped in the jackknife position. Surgical exploration revealed a single erythematous lesion, as shown in Figures 1-3, oozing serosanguinous fluid with visible fistulization into the anal mucosa. Debridement into the anal submucosa showed a total of 22 larvas identified as *C. hominivorax* by morphology. 

**Figure 4:**
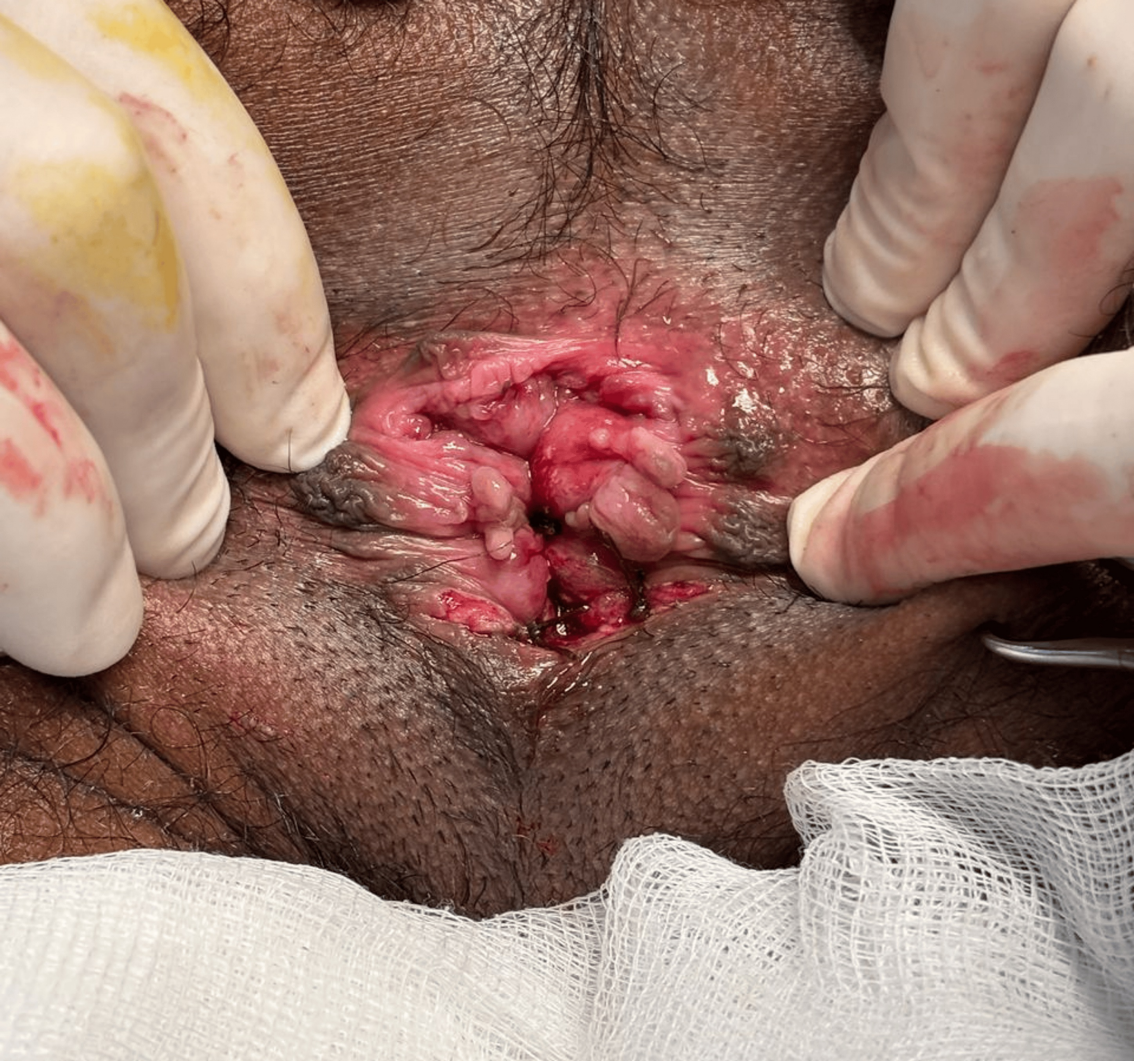
Post-submucosal hemorrhoidectomy wound

Careful dissection and complete extraction of the larvas was ensured. As the hemorrhoidal bundle was severely damaged, we decided to perform a submucosal hemorrhoidectomy, as shown in Figure 4. The area was cleansed with iodine solution and acetic acid. The patient was duly sutured and started on ciprofloxacin 400 mg IV twice a day, continued metronidazole 500 mg IV three times a day, a single oral dose of ivermectin 12 mg, and oral lactulose 30 ml twice a day during his hospital stay. Additionally, we provided supportive analgesia, wound care, and a diet as tolerated. 

The patient improved within 48 hours and was discharged on his third postoperative day. We advised the patient to continue outpatient wound care, maintain regular glycemic control, and take oral antibiotics for the next seven days.

We evaluated the patient at our outpatient surgical service on his 13th postoperative day with complete resolution of symptoms and adequate wound healing.

## Discussion

This case highlights the occupational vulnerability of individuals exposed to unsanitary environments, especially in tropical endemic zones. Significant enabling factors for this case do include the disregard for and lack of education on the proper use of protective equipment.

**Figure 5:**
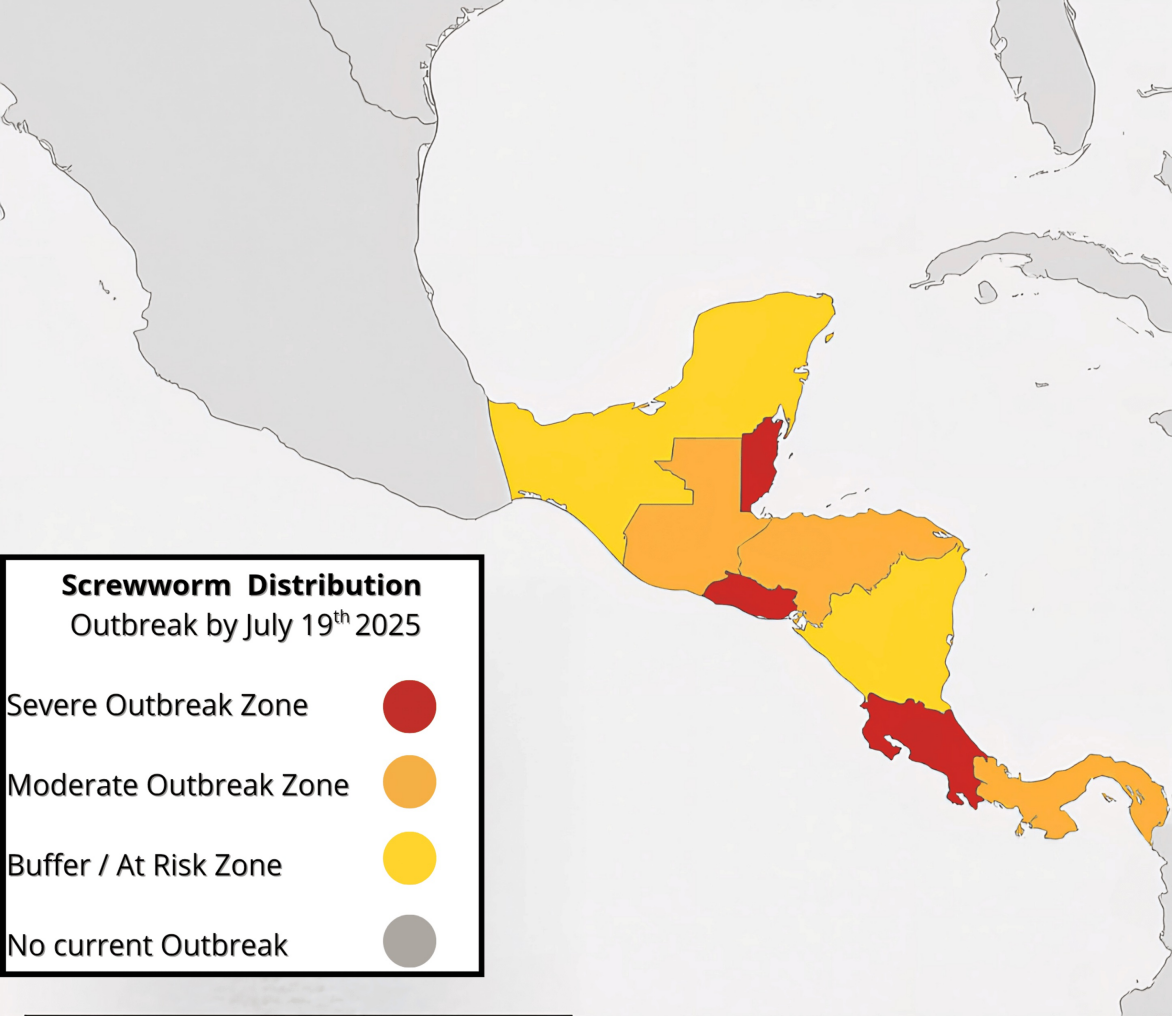
Distribution of The New World screwworm outbreak in Mexico and Central America dated July 19, 2025 Figure created by the authors using data from the US Department of Agriculture's Marketing and Regulatory Programs Business Services, available on the US Department of Agriculture's Animal and Plant Health Inspection Service website [7].

The current outbreak in Central America reported by local and international reports further contextualizes this case presentation, as shown in Figure 5 [7,8]. *C. hominivorax* flies typically perform oviposition directly into open wounds and exposed mucosas of warm-blooded vertebrates. However, as in this case, several factors likely facilitated larval implantation in the anal mucosa, which was already at greater risk due to prolapse hemorrhoids. Such factors include prolonged exposure to fecal sludge, poor hygiene, regular latrine use, and minor skin breaks.

Anorectal myiasis is highly unusual regardless of a limited-resource setting. This is mainly due to the natural motility, sensibility of the region, and regular hygiene. Differential diagnosis includes perianal abscess, thrombosed hemorrhoids, anorectal cancer, and sexually transmitted infections, any of which can obscure an underlying myiasis.

Treatment strategies by involve manual removal of larvas, ivermectin use to minimize the risk of residual infestation, and comprehensive antibiotic coverage to avoid bacterial superinfections. These are of particular importance, especially when involving the gastrointestinal tract, as emphasized in the study by Sunny et al. [9].

Healthcare workers in endemic areas should maintain a high index of suspicion when managing atypical anorectal cases, particularly among vulnerable occupational groups. Additional caution is warranted in suspected immunocompromised patients, as in this case involving a patient recently diagnosed with diabetes. This raises further concerns about factors such as vascular compromise, neuropathy, and impaired immune response, all of which can negatively affect wound healing.

## Conclusions

Anorectal myiasis caused by *C. hominivorax* is rare but possible, particularly among individuals working in poorly sanitized environments. An individualized approach, close attention to the patient’s environmental exposure, and prompt recognition and treatment are essential to prevent complications. Public health interventions should prioritize hygiene education, the enforcement of compliance regulations, and the provision and proper use of personal protective equipment for high-risk workers.
